# Factors Effecting the Total Volatile Organic Compound (TVOC) Concentrations in Slovak Households

**DOI:** 10.3390/ijerph14121443

**Published:** 2017-11-23

**Authors:** Ľudmila Mečiarová, Silvia Vilčeková, Eva Krídlová Burdová, Jozef Kiselák

**Affiliations:** 1 Institute of Environmental Engineering, Faculty of Civil Engineering, Technical University of Košice, Vysokoškolská 4, 04200 Košice, Slovakia; silvia.vilcekova@tuke.sk (S.V.); eva.burdova@tuke.sk (E.K.B.); 2 Institute of Mathematics, Faculty of Science, Pavol Jozef Šafárik University in Košice, Jesenná 5, 04001 Košice, Slovakia; jozef.kiselak@upjs.sk

**Keywords:** TVOC, household, indoor environment, characteristics, dependence

## Abstract

Thirty five Slovak households were selected for an investigation of indoor environmental quality. Measuring of indoor air physical and chemical factors and a questionnaire survey was performed during May 2017. The range of permissible operative temperature was not met in 11% of objects. Relative humidity met the legislative requirements in all monitored homes. Concentrations of total volatile organic compounds (TVOCs) were significantly higher in the apartments than in the family houses. The average TVOC levels in the apartments and family houses were 519.7 µg/m^3^ and 330.2 µg/m^3^, respectively. Statistical analysis confirmed the effect of indoor air temperature, relative humidity and particulate matter (PM_0.5_ and PM_1_) on the levels of TVOCs. Higher TVOC levels were observed also in homes where it is not a common practice to open windows during cleaning activities. Other factors that had a statistically significant effect on concentrations of volatile organic compounds were heating type, attached garage, location of the apartment within residential building (the floor), as well as number of occupants. Higher TVOC concentrations were observed in indoor than outdoor environment, while further analysis showed the significant impact of indoor emission sources on the level of these compounds in buildings. The questionnaire study showed a discrepancy between objective measurement and subjective assessment in the household environment, and pointed to insufficient public awareness about volatile organic compounds (VOCs).

## 1. Introduction

When we count the hours spent sleeping, working or at school, we find that humans spend most of their time in confined spaces. This time amounts to around 90% of the time for people in developed countries, and is even greater for vulnerable sectors of the population (young children, people with weakened health, or seniors). Therefore the indoor air quality (IAQ) is a significant problem that needs to be addressed [1,2]. Personal exposure to VOCs has been investigated in a limited number of studies. Gokhale et al. studied the exposure to VOCs in homes, offices and outdoor in Leipzig (Germany). The highest proportion of personal exposure was from households (42–73%), followed by the outdoor environment (18–34%), and offices (2–38%). Benzene, dodecane, decane, methylcyclopentane, triethyltoluene as well as trichloroethylene prevailed in the outdoor environment, while methyl cyclohexane, triethyltoluene, nonane, octane, tetraethyl toluene, and undecane had the highest concentrations in the offices and whereas a group of terpenoides such as 3-carane, limonene, α-pinene, β-pinene, and the aromatic compounds toluene and styrene had the greatest impact in households [3]. Several other studies [4,5,6] have also pointed to the fact that at home we spend about 60–70% of our time at home. Due to this fact, households are among the most investigated microenvironment in terms of IAQ (resp. the occurrence of VOCs and other pollutants). However, the prevalence of individual VOCs as well as their levels are different and vary not only within countries or cities, but also within households themselves (i.e., in different rooms). The most abundant VOCs the indoor air of Puertollano in Spain were formaldehyde and hexanal, followed by butanal, acetone and acetaldehyde in the indoor air of Puertollano in Spain. On the basis of the indoor/outdoor ratio (I/O ratio) it has been found that the presence of sources in indoor environment is common for limonene, α-pinene, hexanal, formaldehyde, pentanal, acetaldehyde, o-xylene, nodecane, and acetone [7]. The risk level for 93 chemicals polluting the indoor air was calculated in Japan. Formaldehyde, acrolein, 1,4–dichlorobenzene, benzene, tetrachloroethylene and benzo (a) pyrene were ranked in the highest risk category [8]. TVOC concentrations were low, however, about four times higher than in outdoor air, indicating the dominant influence of indoor sources in the established apartments in Melbourne (Australia). Much higher concentrations were observed in new or renovated buildings [9]. High concentrations of VOCs are more often reported in newly built than in already occupied residential buildings [9,10,11,12]. However, VOCs levels in many new residential buildings are declining over time due to the fact that the emission strength of structures and furniture decreases with time [13]. This is supported by several articles [10,12], which have dealt with the long-term course of VOCs concentrations in new residential buildings after the users moved in. In these studies, VOC measurements were repeated for three years with an annual interval. The results showed that most VOC concentrations in new households did not show similar levels to older households after two years. Järnström et al. [11] repeated their VOC measurements in newly completed residential buildings during more than a year with an interval of less than six months and found that the most significant decrease in concentrations occurred during the first six months. In addition, the VOCs coming from the construction phase were replaced by new ones the longer the building was use. The same issue was also addressed by Shin and Jo [14], who monitored the development of VOC concentrations for 24 months with a monthly measurement interval in 25 households in different new residential homes. Both TVOC and VOC concentrations showed a decreasing trend during this period. The average TVOC concentration in the first month of measurement was 881 µg/m^3^ while in the last month was 432 µg/m^3^. Floor coverings/coatings were the most influential indoor source of VOCs followed by cleaning agents, wood paneling/furniture, paints and moth repellents. 

TVOC concentrations in newly decorated rooms ranged from 650 to 690 µg/m^3^ in Hangzhou (China). The characteristics of the emission source were a key factor influencing the concentration. In addition, the levels were influenced by temperature, humidity, time from the end of the decoration to sampling as well as the time at which the windows and doors were closed before sampling while temperature and humidity were less important factors [15]. Noris et al. investigated the impact of building reconstruction in order to reduce the energy demands on the indoor environmental quality (IEQ) in 16 apartments (eight apartments with continuous mechanical ventilation and eight apartments without mechanical ventilation). Their results indicate an improvement in the levels of chemical pollutants including VOCs in the indoor air. In general, apartments with continuous mechanical ventilation showed a more pronounced improvement in IEQ than apartments without this system [16]. The indoor air in 20 new passive houses and 21 new regular houses in Sweden were evaluated by Langer et al. [17] in Sweden. Significant differences in TVOC and formaldehyde concentrations between passive houses and regular houses indicate the presence of substantial TVOC sources in passive houses, while source of formaldehyde may be more pronounced in regular houses. 

In another study, Langer and Bekö [18] investigated the Swedish Housing Stock as well as the relationship between building characteristics and IAQ. Higher concentrations of TVOCs as well as formaldehyde were found in family houses than in dwellings, as well as in dwellings built in between 1955 and 1980 than in new or older apartments. TVOC concentrations were higher in rural areas comparted to cities and in the apartments with natural ventilation compared to those with mechanical ventilation. A significant negative correlation between air exchange and TVOC as well as formaldehyde concentrations reflects the ability of ventilation to reduce the indoor exposure to these compounds. The sum of VOCs in New Delhi (India) ranged from 33.6 to 107.2 µg/m^3^. Higher concentrations were found in the living rooms, followed by kitchens and bedrooms [19]. Dodson et al. [20] investigated the impact of cellars, garages, and common corridors on VOCs in households. Concentrations in garages were 5–10 times higher than the median concentrations of benzene, toluene, ethylbenzene and xylenes in the indoor environment. The ratio of concentrations in cellars/households was significant for methylene chloride, ethylbenzene, m/p-xylene, o-xylene, and summer ratios tended to be higher than winter ratios. Approximately 20–40% indoor concentrations were associated with petrol sources, such as methyl t-butyl ether, benzene, toluene, ethylbenzene and xylenes for households with attached garages. The cellars contributed to approximately 10–20% of the indoor concentrations. For apartments, approximately 5–10% of indoor concentrations were associated with air from corridor. The use of LPG stoves had more significant impact on VOC concentrations than the use of natural gas stoves in Hong Kong [21]. Guo et al. [22] found that formaldehyde concentrations correlated with the age of the building, while this trend was not observed for VOC concentrations. In the study [23], observed that the type of ventilation system and flat placement correlated with formaldehyde concentrations. 

**Figure 1:**
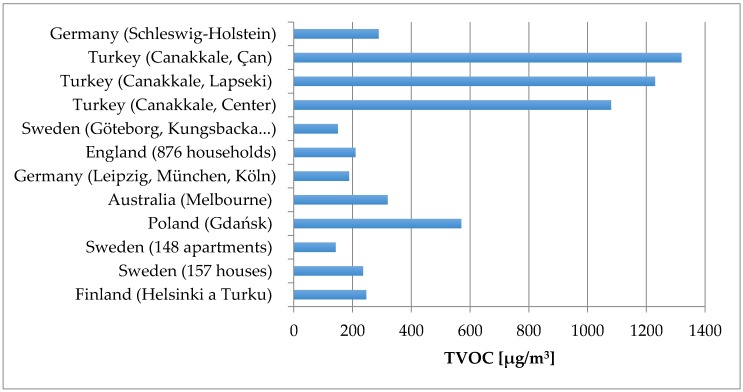
Overview of TVOC levels in homes across the different studies [28,29,30,31,32].

More than 70% of total VOC concentrations in indoor air in Edmonton were assigned to indoor sources where households products were the main contributor, followed by combustion process with tobacco smoke, deodorants, and construction materials. The main outdoor sources of VOCs were the oil and gas industry, transport emissions and biogenic emissions [24]. Building materials were the largest contributor to VOC concentrations in households in Hong Kong, followed by air freshener products, household products, mothballs and painted wood products [25]. Increased concentrations of VOCs in Michigan (USA) were associated with eight sources or activities: the presence of attached garage, recent renovations, older residences, smoking inside, fewer windows and doors, higher CO_2_ concentrations, and a lower level of ventilation [26]. Ohura et al. [27] compared the quality of indoor (living rooms, bedrooms and kitchens) as well as outdoor air in Japan and China during the summer and winter season between 2006 and 2007. Concentrations of many target VOCs (benzene, toluene, ethylbenzene, xylenes and trimethylbenzene) were significantly higher in China than in Japan. Indoor VOC levels in Japan were similar to those outdoor, while in China they tended to be higher. Outdoor sources, including transport and industrial emissions, as well as human activity, were significant sources of VOC pollution. As can be seen, the results from different countries, cities differ in their conclusions, identified VOC or TVOC levels. Overview of TVOC levels in residential buildings in different countries/cities is illustrated in Figure 1.

The main contribution of the presented research work is finding the dependence between indoor air factors and building characteristics. Determination of TVOC occurrence and analysis of the main factors affecting the TVOC levels in Slovak households are also important outputs due to the fact that this type of data are insufficient. Only limited number of researches were carried out in residential environment in our country [33].

## 2. Materials and Methods

### 2.1. Objective Measurement

Experimental measurements focused on determination of TVOC levels were performed in 35 households—18 apartments (B1–B18) and 17 family houses (D19–D35)—in May 2017 (Spring). The air temperature (T_a_), relative humidity (RH), mean radiant temperature, concentration of total volatile organic compounds (TVOC), and concentration of particulate matters (PM_0.5_–PM_1_) were monitored in each household during 1 h. Measurement of TVOC concentrations were also performed during 1 h in outdoor environment (at a distance approximately 3 m from the building) for determination of I/O ratio. The air temperature as well as relative humidity was monitored with data logger TESTO 175-H2 (Testo, Inc., Lenzkirch, Germany). The concentrations of PM were determined using a HANDHELD 3016 IAQ (Lighthouse Worldwide Solutions, Inc., Fremont, CA, USA). TVOC concentrations were measured with photoionization detector with UV lamp—ppbRAE 3000 (RAE Systems, Inc., San Jose, CA, USA). Data from measuring devices were recorded in a minute interval. Mean radiant temperature in three heights were measured using a Vernon-Jokl globe thermometer (d = 0.01 m) according to EN 7726. Operative temperatures (T_o_) were calculated also according to EN 7726 [34]. This parameter was calculated for comparison with the recommended and permissible values for indoor environment in Slovak legislation. Basic information about measuring devices are summarized in Table 1. Measuring devices were placed in the center of the room in the height approximately 1.1 m above the floor (breathing zone). Household users were asked not to ventilate approximately 2 h before the measurement in the selected room. Before the measurement the household regime was not influenced in any other activities to determine the actual state of IAQ in the selected households. Measurements were carried out before the noon in living rooms during normal operation, but windows and doors were closed throughout the measuring period. Living rooms were chosen for measurement due to the fact that it is a central room of apartments given that all members of household meet in this area and spend time together here. 

### 2.2. Subjective Measurement

Comfort assessment is an important tool for gathering information from building users about their subjective perception of individual indoor environmental quality factors, including air quality, which was also used during this research. Our questionnaire (Appendix A) was based on a study of the current scientific information about occurrence of VOCs in indoor environment and consisted from five main parts: basic information about the building (residential building/family house), basic information about the place of measurement (selected room), information about household regime (smoking, use of fragrances, etc.), specification of IEQ problems (odor, dust, temperature instability, etc.), and occurrence of sick building syndrome symptoms (SBS). The aim of questionnaire survey was to obtain the most accurate information not only from the construction point of view, but also about households regime, that were helpful in analysis of the main factors affecting the TVOC levels in Slovak households.

### 2.3. Statistical Analysis

Results from experimental measurement as well as from questionnaires were statistically evaluated using the R software (The R Foundation for Statistical Computing, Vienna, Austria). All procedures can be found in package vegan (except the Wilcoxon rank sum test). Ordinations were plotted with non-metric multidimensional scaling (NMDS) [35]; using the default settings of vegan’s “metaMDS” procedure and “ordiellipse”, which adds ellipses enclosing all points in the group (ellipsoid hulls) or ellipses of standard deviation, standard error or confidence areas. This allows us to visualize the level of similarity of individual cases of a dataset. It uses adequate dissimilarity measures. Since variances for “TVOC” were large, we have used the Bray Curtis distance (or Sorensen distance), which is a normalization method commonly used in environmental science fields. It has a nice property, that for positive coordinates its value is between zero and one. Procedure “envfit” fits vectors of continuous variables and centroids of levels of class variables (defined as factor in R). The direction of the vector shows the direction of the gradient, and the length of the arrow is proportional to the correlation between the variable and the ordination. It can also assess the significance of the variables using permutation tests. Function stress plot draws a Shepard plot where ordination distances are plotted against community dissimilarities, and the fit is shown as a monotone step line. In addition, stress plot shows two correlations like statistics of goodness of fit. The correlation based on stress is R^2^ = 1 − S^2^. The “fit-based R^2^” is the correlation between the fitted values and ordination distances, or between the step line and the points. This should be linear even when the fit is strongly curved and is often known as the “linear fit”. These two correlations are both based on the residuals in the Shepard plot, but they differ in their null models. In linear fit, the null model is that all ordination distances are equal, and the fit is a flat horizontal line.

Factor variables (year of construction, type of heating, thermal insulation, etc.) and their impact on TVOC levels were then (after finding their significance using permutation tests in the envfit procedure) evaluated using the Wilcoxon rank sum test. The Wilcoxon rank-sum test is a nonparametric alternative to the two sample *t*-test which is based solely on the order in which the observations from the two samples fall. The exact *p*-value is determined from the distribution of the Wilcoxon Rank Sum Statistic W. Moreover it returns a “difference in location” measure, which tell as the data differ [36].

### 2.4. Characteristics of Investigated Households

The basic information about selected objects are shown in Appendix B—Table 2 and Table 3, Table A1 and Table A2 (Heating type: CH—central heating, SF—solid fuel heating, G—gas boiler, O—others (electric heater, heat pump); Main building material: CO—combination of materials (burnt brick + porous concrete), PC—porous concrete, CS—clay + stone, BB—burnt brick; Flooring: L—laminate flooring, C—carpet, O—others (PVC, brick, wooden parquet)). Natural ventilation is used in all selected homes. Central heating and plastic windows were a common feature in all apartments, except for B13 and B14, where heating is ensured by electric heaters. Plastic windows were also dominated in family houses. The most prevalent type of heating in family houses was the gas boiler used by 76% of households. 60% of the buildings were thermally insulated, while all residential buildings and 82% of family houses were insulated with polystyrene. Wood-fibre or mineral wool was used for thermal insulation in other family houses. The furnishing in each households were almost identical (living rooms) and consisted from TV wall, sofa, coffee table or smaller cabinets. In some cases (larger floor area of monitored room), the living room was connected to the kitchen. 

The predominant materials used in the selected rooms were: particleboard (furniture), laminate flooring, and dispersion paint on walls and ceilings. The monitored room was renovated in less than 30 weeks before the measurement only in four households (three apartments, one family house). The garage has 59% of family houses, while 35% of them have the garage attached to the house with direct entry. Gasoline, paint, solvents, etc., were stored in the garage in all cases. Pets were present in 26% of households. Smoking indoors and problems with mould were confirmed also in 26% of households. 43% of households use fragrances such as air fresheners, scented candles, aromatic sticks or oils, etc. The printer is used in the living room by 11% of households. Newspaper/magazines and toys are stored in living room by 77% and 29% of respondents. Mothballs, which are a significant source of VOCs, are used only by 6% of households. Art supplies are used in the chosen room in 11% of objects. The detergents do not use only 6% of households, on the contrary polishes/waxes for floors or furniture use 23% of households. Frequency of cleaning varied between objects. 74% of the monitored objects has open windows during cleaning activities.

## 3. Results and Discussion

### 3.1. Subjective Measurement

**Figure 2:**
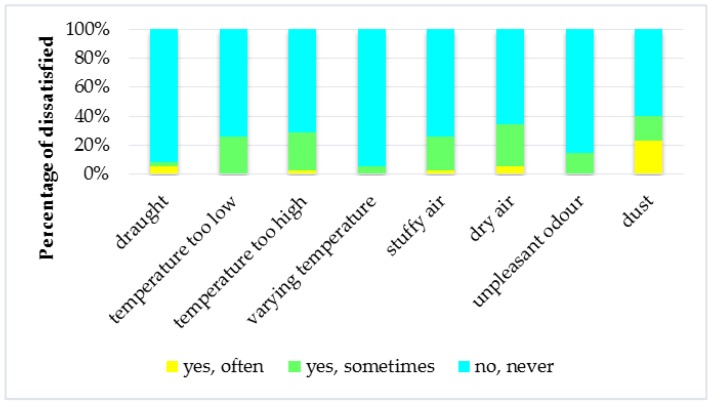
Problems with factors affecting the IEQ.

**Figure 3:**
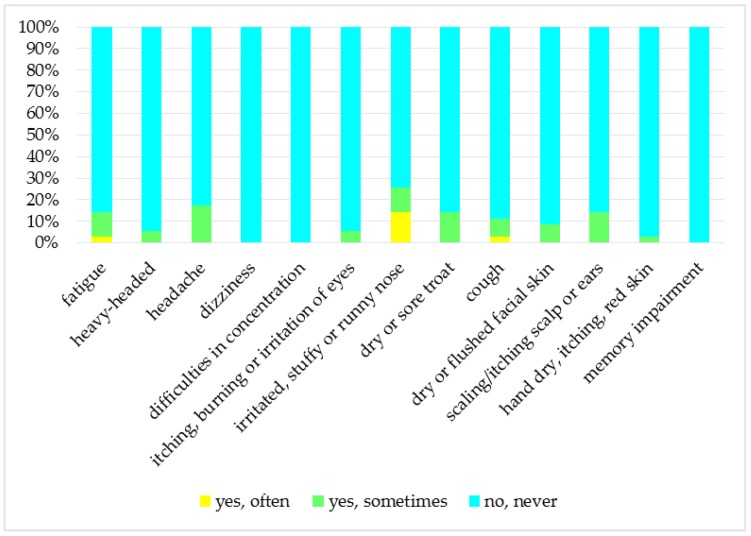
Prevalence of SBS symptoms.

Questionnaires were filled by 35 respondents (57.1%—women). 51.4% of persons were aged 20 to 35, 25.7% aged 36–50, 11.4% aged 51–55, and 11.4% over the age of 66. Smokers represent 31.4% of respondents. The largest group (45.7%) consisted of households with the number of occupants ≤2 people followed by a group with 3–4 people (34.3%) and a group with number of households users greater than 4 (20%). 85.7% of respondents spend more than 12 h a day at home. Only 8.6% of people completing the questionnaire have a doctor diagnosed asthma and 34.3% have a doctor diagnosed allergy. Figure 2 illustrates the percentage of households that have a problem with factors affecting of IEQ as a draught, temperature too low, temperature too high or varying temperature, stuffy air, unpleasant odour, and dust. As can be seen, the biggest problem in households was dry air and dust. In case of SBS symptoms prevalence, it can be concluded that in households their occurrence is rare (Figure 3). 

The most common symptom was irritated or stuffy nose. Comparing the results from monitoring of physical and chemical factors with the results of the questionnaire survey, it was found that users rated individual factors of IEQ more mildly than usual in other types of environment (for example in workplace).

### 3.2. Objective Measurement

**Figure 4:**
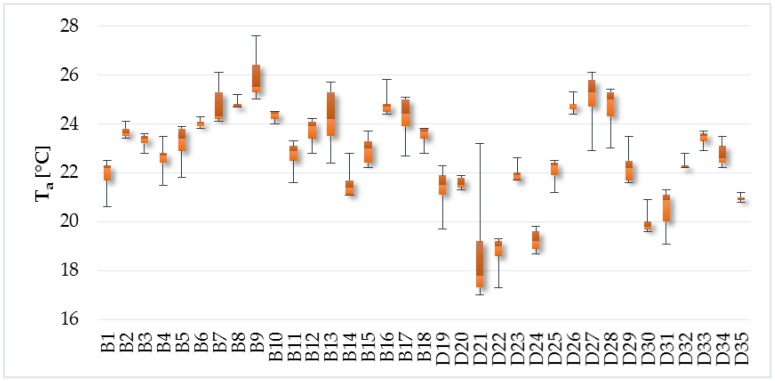
Indoor air temperature.

**Figure 5:**
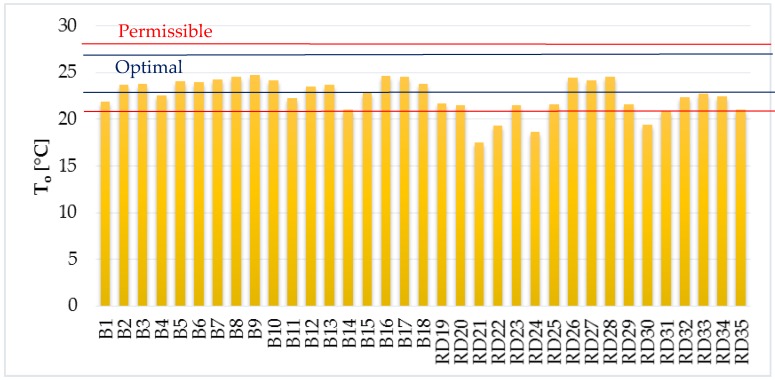
Operative temperature.

The average, minimum, and maximum air temperature measured in the residential buildings were 23.7 °C, 20.6 °C, and 27.6 °C, respectively (Figure 4). The air temperatures in the family houses were slightly lower. The average, minimum, and maximum air temperature in the family houses were 21.8 °C, 17 °C, and 26.1 °C, respectively. The optimal operative temperature for activity class 0 (peaceful relaxation, relaxed seating (resting, watching the TV)) should be in the range of 22–26 °C and permissible operative temperature should be in the range of 20–27 °C for May according to Decree no. 210/2016 [37]. As can be seen from Figure 5, the range of optimal operative temperature was not reached in 37% of the objects. The range of permissible operative temperature was not reached in 11% of the objects. The average, minimum, and maximum operative temperature in the apartments was 23.6 °C, 21.1 °C, and 24.7 °C, respectively. The average, minimum, and maximum operative temperature in the family houses were 21.5 °C, 17.5 °C, and 24.5 °C, respectively.

**Figure 6:**
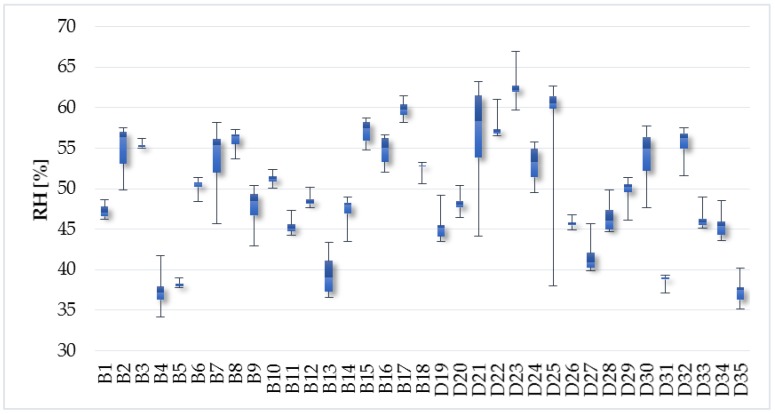
Relative humidity.

The average relative humidity was 49.9%, minimum was 34.2%, and maximum was 61.5% in the apartments. There was no significant difference in the family houses compared to the apartments. The average, minimum, and maximum relative humidity in the family houses was 49.6%, 35.2%, and 66.9%, respectively (Figure 6). Permissible relative humidity in the indoor environment should be in the range of 30–70% [37]. This requirement was fulfilled in all households.

**Figure 7:**
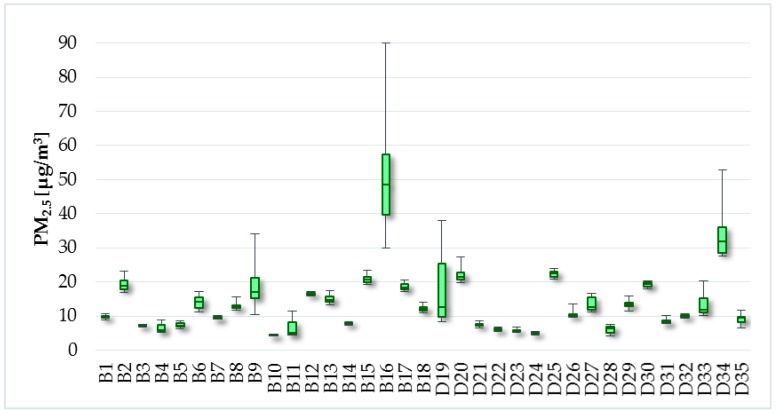
PM_2.5_ concentrations.

**Figure 8:**
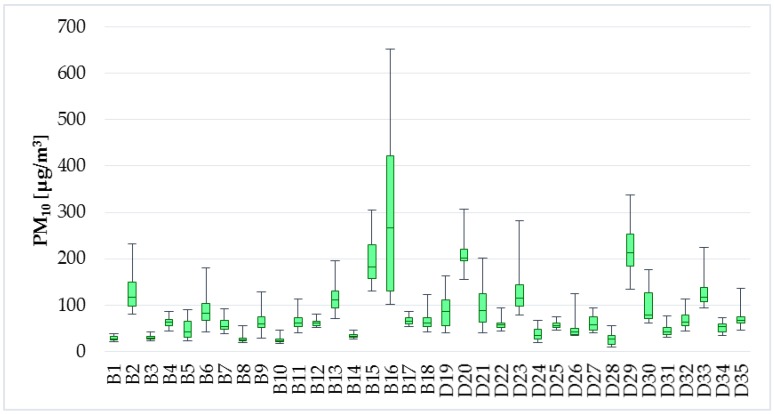
PM_10_ concentrations.

The limit value for PM_10_ concentrations is determined at 50 µg/m^3^ for 24 h exposure in the Decree no. 210/2016 [37]. The results from measurement in the selected households cannot be compared with this legislative requirement since the measurement not lasted 24 h. However, the intention for measurement of PM levels was to find out whether there is dependence between the TVOC concentrations and individual PM fractions. The average concentrations of PM_0.5_-PM_1_-PM_2.5_-PM_5_-PM_10_ were in the apartments and family houses as follows: 5.4; 5.8–8.1; 8.3–14.3; 13.1–44.1; 44.5–81.0; 87.8 µg/m^3^. Levels of PM_2.5_ and PM_10_ are illustrated in Figure 7 and Figure 8.

**Figure 9:**
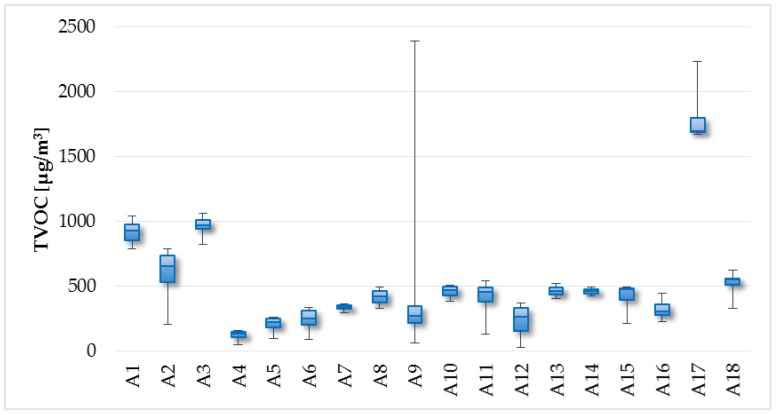
TVOC concentrations—the apartments.

**Figure 10:**
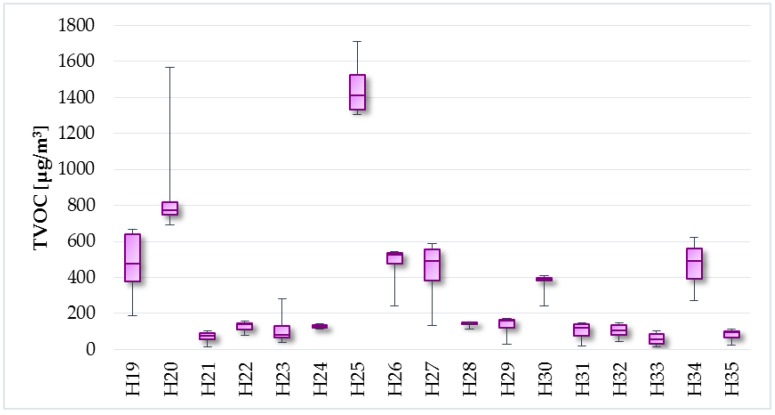
TVOC concentrations—the family houses.

The measured values of TVOC concentrations in individual objects are illustrated in Figure 9 and Figure 10. The average, minimum, and maximum TVOC concentrations in the apartments were 519.7 µg/m^3^, 28 µg/m^3^, and 2393.5 µg/m^3^, respectively. TVOC levels were slightly lower in the family houses, with average value of 330.2 µg/m^3^, minimum value of 13.5 µg/m^3^, and maximum value of 1712 µg/m^3^. The recommended value for TVOC concentrations is 200 µg/m^3^ according to Mølhave [38]. This value was exceeded in 69% of households (94% of apartments and 41% of family houses). 

**Figure 11:**
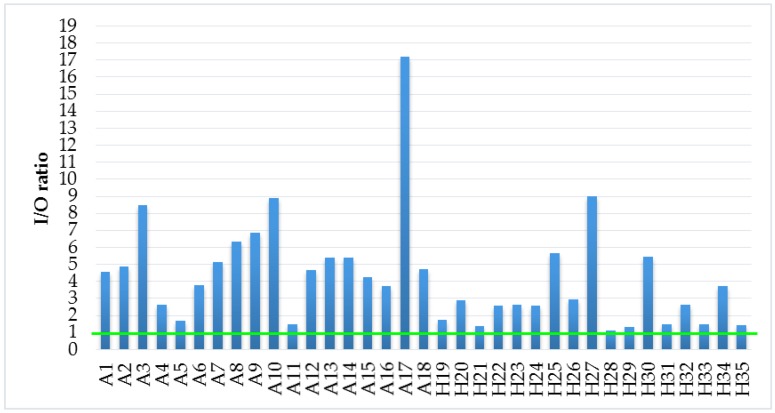
I/O ratio of TVOC levels.

As mentioned above, the I/O ratio was calculated for all selected households. The average, minimum, and maximum outdoor TVOC levels were 108.8 µg/m^3^, 38.8 µg/m^3^, and 292 µg/m^3^, respectively. If I/O ratio is less than 1, it can be concluded that outdoor air is the main source of TVOC in indoor environment. As can be seen in Figure 11, this case did not occurred in selected households and it can be stated that the sources of TVOC pollution originate from the indoor environment. 

### 3.3. Statistical Analysis

**Figure 12:**
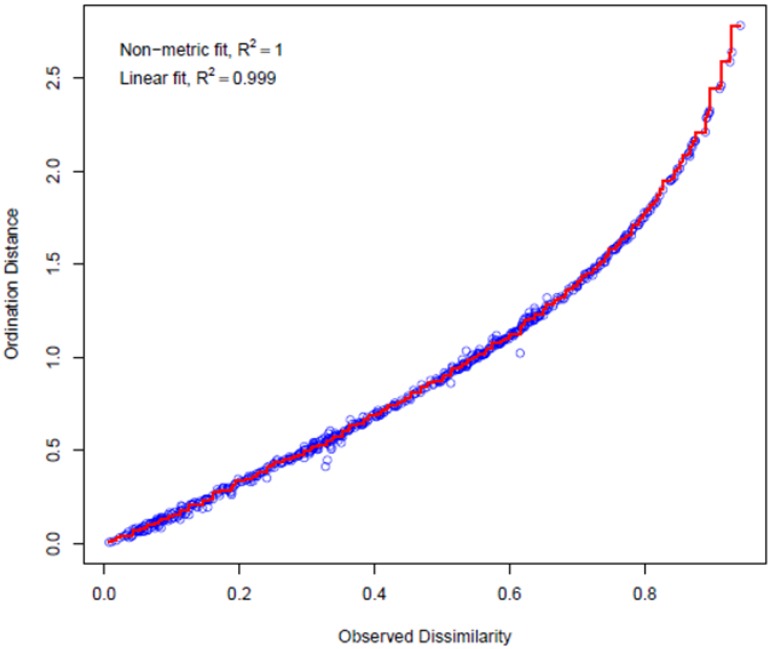
Shepard plot.

The relationship between continuous variables (air temperature, relative humidity, and PM_0.5_–PM_10_), factor variables (factors summarized in Appendix B—Table A1, Table A2, Table A3 and Table A4), and TVOC concentrations measured in the indoor air of individual households was analyzed using statistical methods described in the Section 2.3. The dependence of similarity on the selected metric (Bray Curtis metric) is plotted using the Shepard diagram (Figure 12). In this case, the match is excellent. Table 2 lists only those factors and variables that shown a statistically significant effect on TVOC levels in households and Table 3 lists factors that were additionally tested using the Wilcoxon rank sum test. 

**Figure 13:**
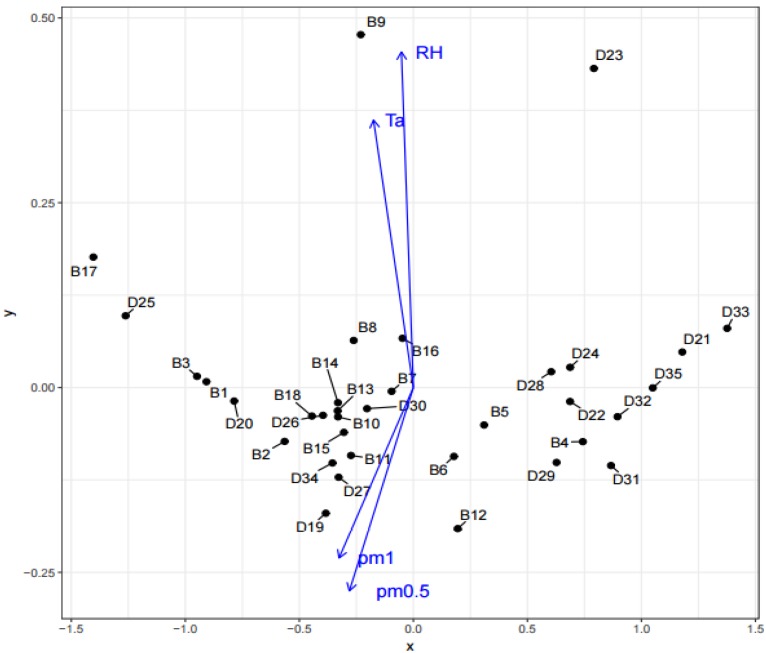
Dependence of continuous variables.

The results from analysis of continuous variables are shown in Figure 13. Objects in the direction of variables arrow are objects in which the TVOC levels positively correlated with given variable. Objects in the opposite direction to the variables arrow direction are objects in which the TVOC levels negatively correlated with a given variable. However, it should be emphasized that this is a case of partial correlation and thus several factors could have impact on the TVOC concentrations in the given object. Relationships were confirmed between TVOC and temperature, relative humidity, PM_0.5_ as well as PM_1_. Effect of environment conditions such as temperature and relative humidity on TVOC levels in buildings has been the subject of numerous studies [1,15,39,40]. Negative correlation between TVOC and PM_0.5_ as well as PM_1_ was also confirmed in the study [40].

**Figure 14:**
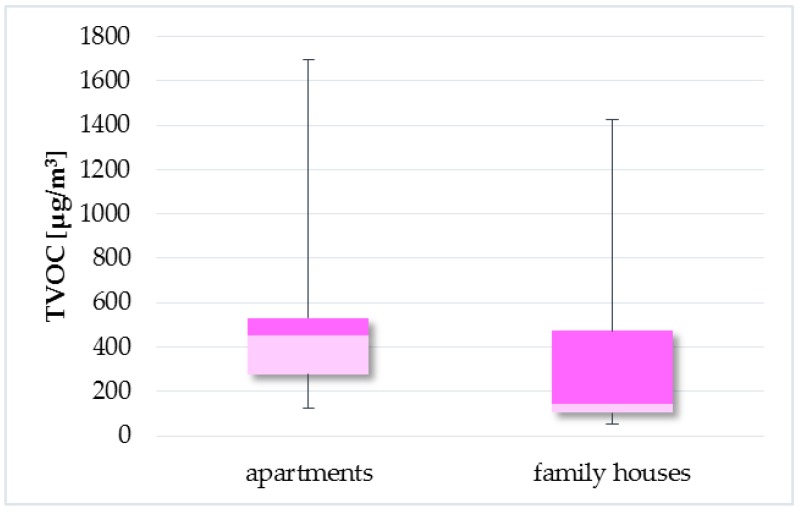
Boxplot of total medians—the apartments and family houses.

As can be seen in Figure 14, the TVOC concentrations were significantly higher in the apartments than in the family houses, which was confirmed by the Wilcoxon rank sum test (*p* = 0.4865). On the contrary, studies [18,41] show higher TVOC levels in the family houses than in the apartments. 

**Figure 15:**
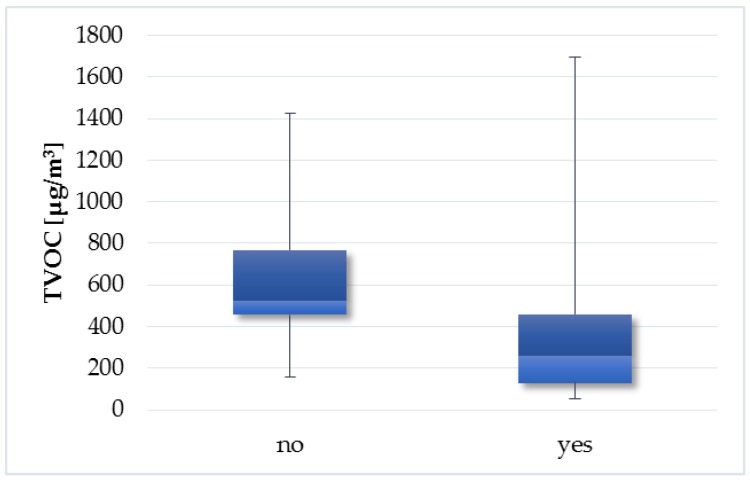
Boxplot of total medians—windows opening during cleaning activities.

**Figure 16:**
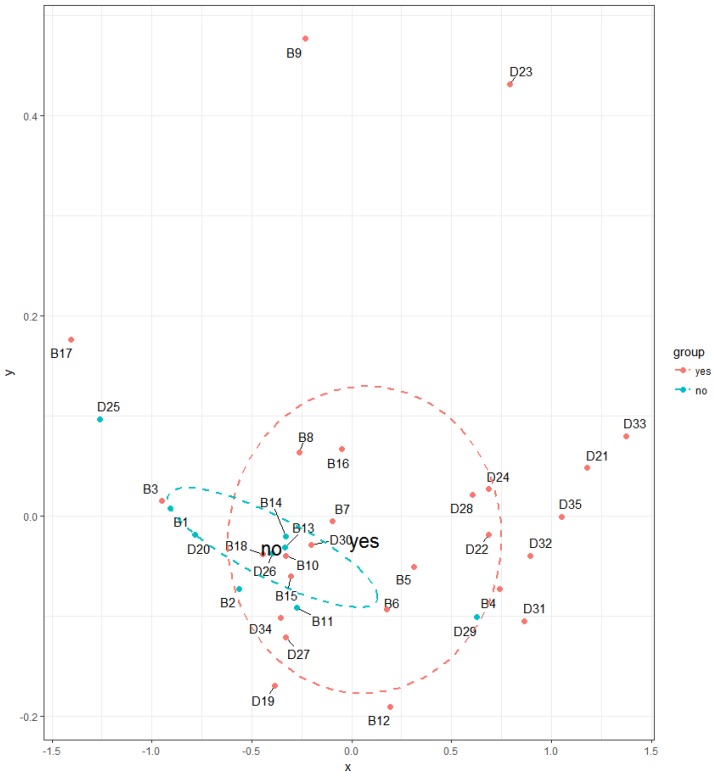
NMDS visualizing the level of similarity for the factor—windows opening during cleaning activities.

**Figure 17:**
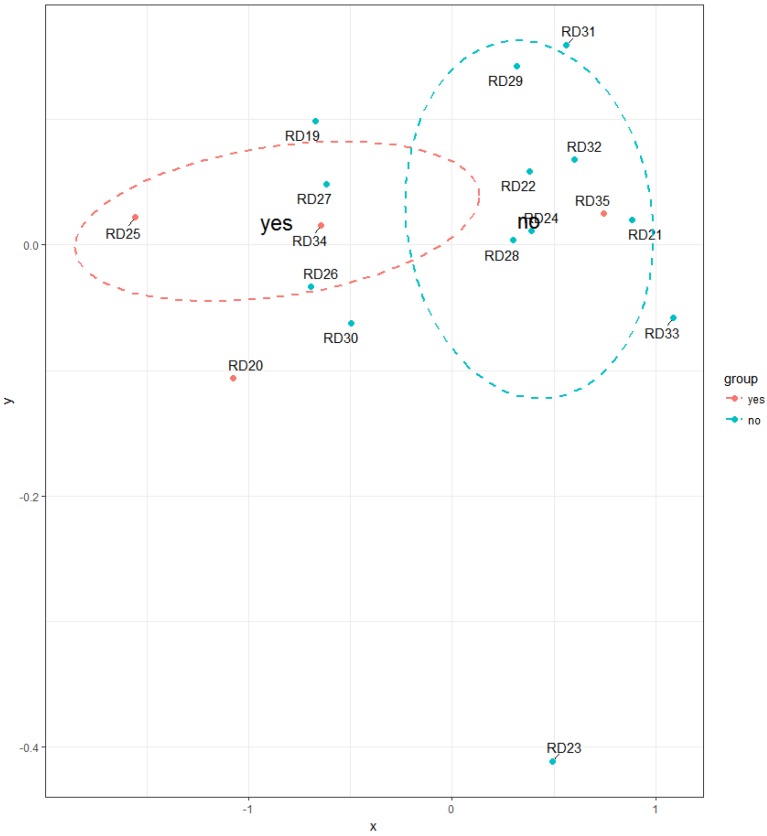
NMDS visualizing the level of similarity for the factor—attached garage.

**Figure 18:**
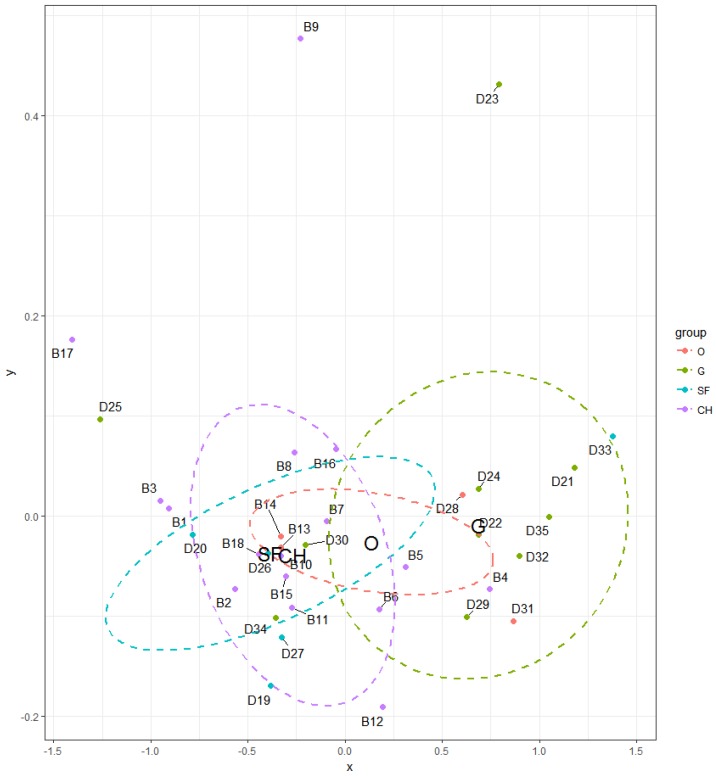
NMDS visualizing the level of similarity for the factor—type of heating.

**Figure 19:**
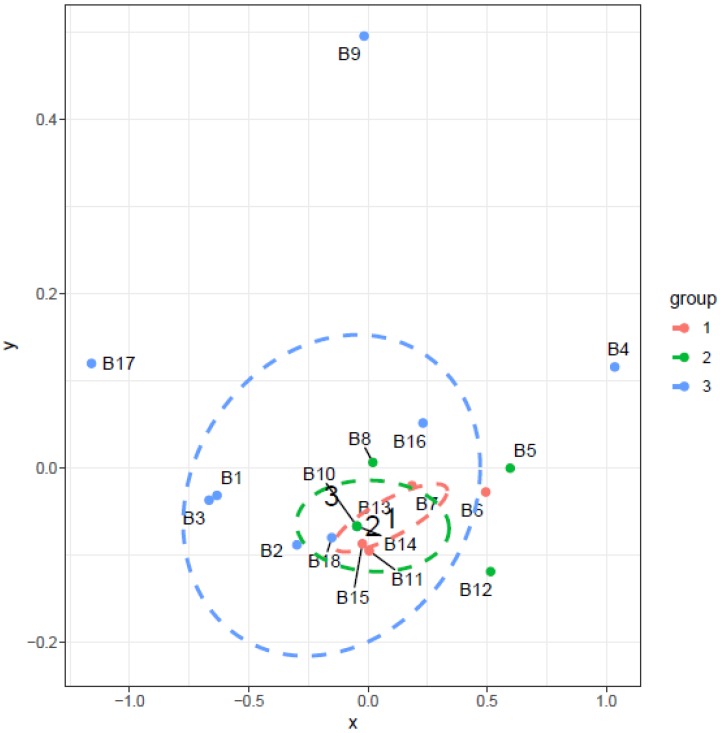
NMDS visualizing the level of similarity for the factor—floor of the apartments.

**Figure 20:**
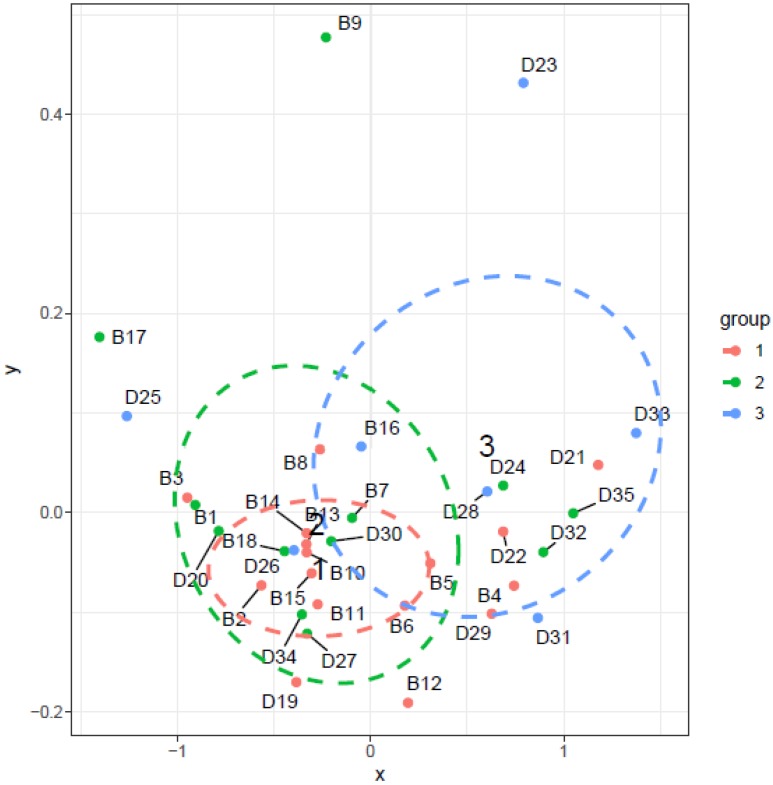
NMDS visualizing the level of similarity for the factor—windows opening during cleaning activities.

**Figure 21:**
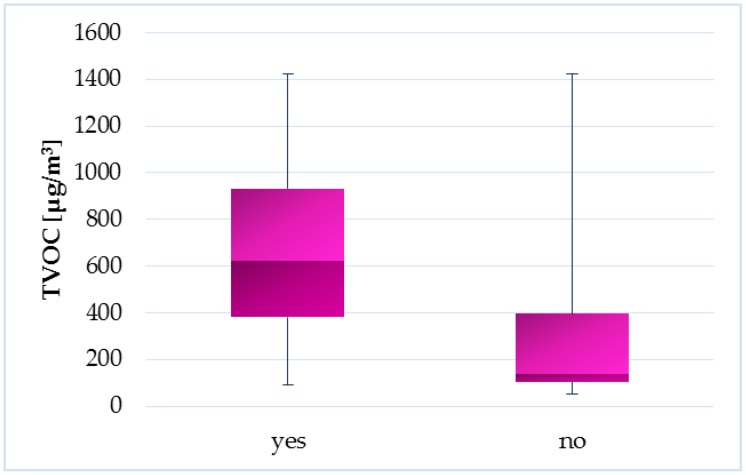
Boxplot of total medians—attached garage.

Households, where it is common practice to open windows during cleaning activities, have statistically significants lower TVOC concentrations (*p* = 0.01019) compared to households where the windows do not open during cleaning (Figure 15). Cleaning products belong to the important source of indoor VOC emissions [42,43,44,45]. Therefore, impact of this factor on TVOC concentrations in Slovak households is reasonable. Figure 16, Figure 17, Figure 18, Figure 19 and Figure 20 visualize the level of similarity for a given factor. Elipses are drawn around the standard deviation of group dispersions in the ordination plots. That is a measure of spred of the data. The groups are created according to a given factor, and if they are without penetrations, there is a clear division into groups. If there is visible crossover between the groups, it means that the factor not only in itself breaks down the observed groups. The TVOC concentrations were significantly higher (*p* = 0.05084) in the family houses with attached garage (Figure 21). In all cases, direct access to the house was allowed from attached garage, which allowed the migration of organic pollutants into the household environment. The fact that high concentrations of VOC present in garages (mainly due to car emissions) can affect indoor concentrations has been confirmed in several studies [46,47,48,49,50,51,52,53]. The VOCs composition in garages reflects the compounds expected for gasoline vapours as well as compounds associated with paints, solvents, cleaning agents and other materials used and stored in households, garages and vehicles [53].

**Figure 22:**
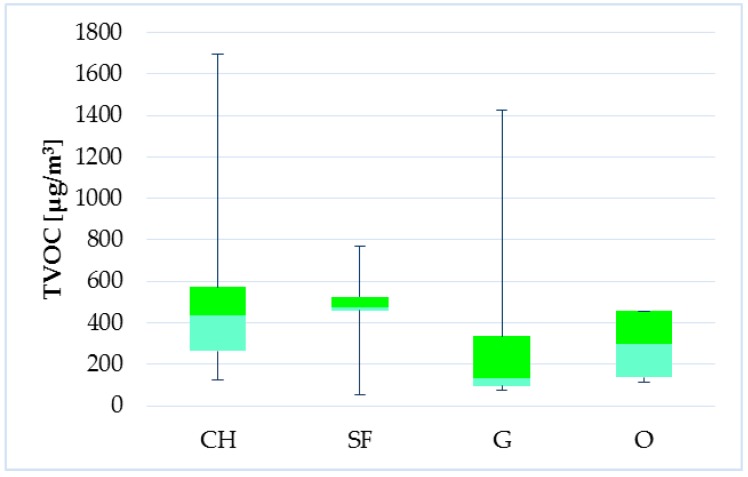
Boxplot of total medians—type of heating.

The influence on TVOC levels in indoor environment of selected households also had a type of heating but in lesser extent (*p* = 0.09091). However, the difference between central heating (CH) and solid fuel heating (SF) was not significant (*p* = 0.7798) and also the difference between gas boiler (G) and other types of heating (O—electric heater, heat pump) was not significant (*p* = 0.4535). During comparing central heating and solid fuel heating versus other types (G + O) were confirmed the effect on TVOC levels (*p* = 0.01461). It follows that higher concentrations of TVOC were observed in household with central heating as well as with solid fuel heating than in other households (Figure 22). However, this factor needs to be further examined. 

**Figure 23:**
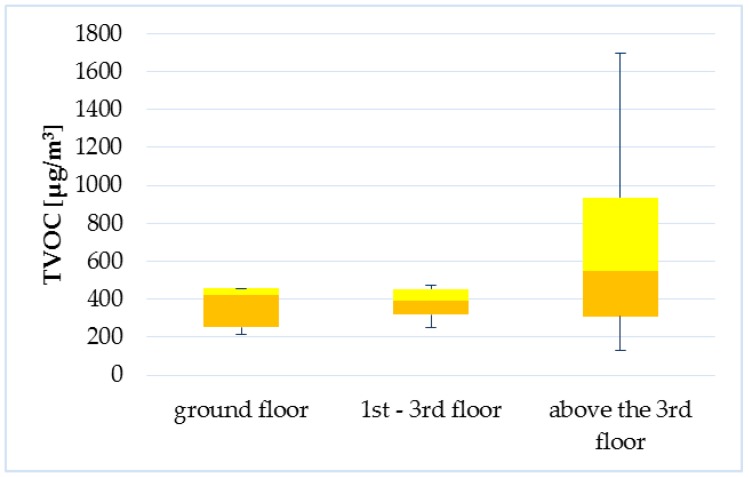
Boxplot of total medians—floor of the apartments.

The apartments were divided into three categories: ground floor (1), 1–3 floor (2), and above the 3rd floor (3). The initial analysis pointed to the significant influence of the floor on TVOC levels in the apartments. Because the difference in TVOC medians between the first two categories was small, two categories were tested in additional analysis: lower than 3rd floor and above 3rd floor. This test revealed a significant difference between the TVOC levels on the lower floors compared to the higher floors (*p* = 0.05675), while Figure 23 is shown the higher floor the higher levels. The influence of this factor can be explained by chimney effect. The opposite phenomenon was observed in a study of Jo et al. [54] where higher concentrations of methyl-tertiary butyl ether, benzene, and toluene were observed in lower-floor apartments than on higher floors. 

**Figure 24:**
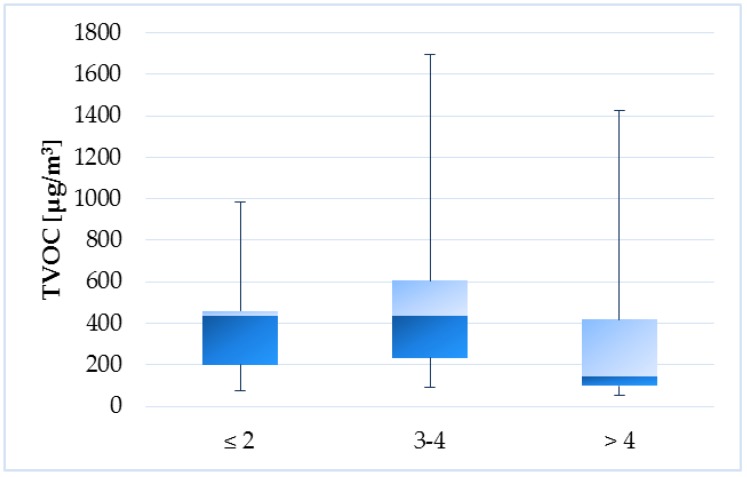
Boxplot of total medians—number of users.

For the number of users, the objects were divided into the following groups: ≥2 users (1), 3–4 users (2), >4 users (3). Figure 24 illustrates the fact that TVOC levels were significantly lower (by 67%) in households with greater number of users. The number of household users have a statistically significant effect on the TVOC concentrations in the indoor air (*p* = 0.04895) but closer testing only confirmed the less significant effect of the higher number of users to lower TVOC levels (*p* = 0.09933). On the other hand, Guo et al. [22] observed higher TVOC levels in household with higher number of occupants, which explained by the fact, that households with higher number of users frequently used air fresheners or cleaning products than households with lower number of users. Since human activity is a significant source of VOCs in the indoor environment, it is logical that the number of users will affect the TVOC levels. In our study, this may be a combination of several factors. Households with a larger number of users mostly had a larger floor area and therefore there was probably a better dilution of contaminants, which would explain the observed lower TVOC levels. 

## 4. Conclusions

The recommended value of TVOC for indoor environment (200 µg/m^3^) was exceeded in 69% of households (94% of the apartments and 41% of the family houses). Based on a comparison of indoor and outdoor TVOC levels it was found that the indoor environment was a significant source of the pollution. The range of optimal operative temperature was not reached in either of the monitored objects. On the other hand, requirement for relative humidity in indoor environment was fulfilled in each household. The statistical evaluation of the data revealed a significant influence of the indoor air temperature, relative humidity and particulate matter concentrations (PM_0.5_ and PM_1_) on the TVOC levels. TVOC concentrations were significantly higher in the apartments than in the family houses as well as in the households where it is not a common practice to open windows during cleaning activities. The type of heating belonged to the factors that play an important role in the occurrence of organic compounds in the indoor environment. This study also confirmed that attached garage contribute to the higher levels of TVOC in the home. Higher concentrations were found in the apartments located on higher floors. The prevalence of SBS symptoms was rare and mostly reported symptoms were irritated or stuffy nose. Only 40% of household users knew about volatile organic compounds and about the possible risks that exposure to these substances might cause. However, only 13% of respondents takes into account VOC emissions when choosing building materials, paints, furniture etc. It follows that knowledge of this issue does not guarantee an aware approach. Nevertheless, the results of this study point to the need to inform the public not only in relation to the basic information on these compounds but also on the possibilities of improving indoor air quality, respectively indoor environmental quality as a whole. 

## Figures and Tables

**Table 1:** Basic information about measuring instruments.

Name	Unit	Range	Accuracy
TESTO 175-H2—T_a_, T_e_	°C	−20~+70	±0.5
TESTO 175-H2—RH	%	0~100	±3
HANDHELD 3016 IAQ	µm	0.3~10	50% @ 0.3 μm; 100% for particles >0.45 μm (per ISO 21501-4)
Vernon-Jokl globe thermometer	°C	0~50	-
ppbRAE 3000	ppm	1~10,000	±3% in calibration point

**Table 2:** Results from envfit.

Variables	r^2^	*p*
Number of users	0.1689	0.04895 *
Heating type	0.1814	0.09091
Windows opening during cleaning activities	0.1555	0.01798 *
Floor of the apartments	0.2741	0.06397
Attached garage	0.2148	0.05195 .
Temperature	0.1614	0.04496 *
Relative humidity	0.2088	0.01698 *
PM_0.5_	0.1544	0.07493
PM_1_	0.1595	0.06893

Significance level: “*” 0.05; “.” 0.1.

**Table 3:** Results from Wilcoxon rank sum test.

Variables	W	*p*
The apartments vs. family houses	213	0.04865 *
Number of users smaller than 4	130	0.09933 .
Heating—SF and CH	219	0.01461 *
Windows opening during cleaning activities	50	0.01019 **
Higher than 3rd floor in the residential building	22	0.05675 *
Attached garage	41	0.05084 *

Significance level: “**” 0.01; “*” 0.05; “.” 0.1.

## Abbreviations

The following abbreviations are used in this manuscript:

TVOC	Total volatile organic compounds
VOCs	Volatile organic compounds
IAQ	Indoor air quality
I/O	Indoor/outdoor ratio
IEQ	Indoor environmental quality
LPG	Liquefied petroleum gas
CO_2_	Carbon dioxide
T_a_	Indoor air temperature
RH	Relative humidity
PM	Particulate matters
UV	Ultraviolet
T_o_	Operative temperature
SBS	Sick building syndrome
NMDS	Non-metric multidimensional scaling
B1–B18	Apartments 1–18
D19–D35	Family houses 19–35
SF	Solid fuel heating
CH	Central heating
G	Gas boiler
O	Other types of heating
CO	Combination of materials
PC	Porous concrete
CS	Clay + stone
BB	Burnt brick
L	Laminate flooring
C	Carpet

## Appendix A

Sample questionnaire (translated from Slovak)

Information about the residential building and the apartment

1. Year of construction of your residential building?
2. Was your residential building renovated?
3. If yes, when and what this renovation included?
4. Is your residential buildings thermally insulated?
5. If yes, what kind of thermal insulation?
6. On which floor is your apartment?
7. What is the total area of your apartment?
8. What is the floor area of the room where the measurement is carried out?
9. What is the ceiling height in the room where the measurement is carried out?
10. What is the number, size (width × height), window material in the room where the measurement is carried out?
11. What is the number, size (width × height), door material in the room where the measurement is carried out?
12. What is the orientation of the windows in this room?
13. What is the traffic density in the immediate vicinity of your apartment?
14. Does your apartment any damage due to moisture?
15. Do you have mould problems in your apartment?
16. Do you have mould problems in the room where the measurement is carried out?
17. What type of heating do you have in your apartment?
18. What type of ventilation do you have in your apartment?
19. What type of finishing (paint, wallpaper) is on the walls in the room where the measurement is carried out?
20. What type of floor is in the room where the measurement is carried out?
21. What type of furniture is in the room where the measurement is carried out?
22. How many plants there are in the room where the measurement is carried out?
23. Do you smoke indoors?
24. Do you use a moth balls in the room where the measurement is carried out?
25. Do you have newspapers or magazines in the room where the measurement is carried out?
26. Do you use air fresheners, scented candles, aromatic sticks, aromatic oils, etc.?
27. Do you use a printer or scanner in the room where the measurement is carried out?
28. Do you store toys in the room where the measurement is carried out?
29. Do you use art supplies in the room where the measurement is carried out?
30. Do you use cleaning agents for cleaning?
31. Do you use polishes or waxes for floors or furniture?
32. How often do you clean the room where the measurement is carried out?
33. Do you have open windows during cleaning?
34. Was your apartment renovated?
35. If yes, when was your apartment renovated?
36. What did this renovation included? (new plasters + paint, new wallpapers, new furniture, new floors...)
37. Have you ever heard about volatile organic compounds (VOCs)?
38. When purchasing building materials, paints and furniture, does the information on VOC emissions from the product play a role in your decision?

Information about family house—following questions were added to previous questionnaire

39. What is the main building material used to build your house?
40. Do you have a garage?
41. Is the garage connected to the house?
42. Is it possible to enter the house from the garage directly through the door?
43. Do you store paint, solvents, or gasoline in the garage?

Evaluation of IEQ

1. Gender
2. Age
3. Smoking
4. How many people live in your household?
5. How much time do you spend at home per day?
6. Do you have a pet?
7. Do you have a problem with one of these factors at home? (yes, often/yes, sometimes/no, never)
  (a) Draught
  (b) Temperature too low
  (c) Temperature too high
  (d) Varying temperature
  (e) Stuffy (bad) air
  (f) Dry air
  (g) Unpleasant odour
  (h) Dust
8. Have you ever been diagnosed by a doctor to have asthma?
9. Have you ever been diagnosed by a doctor to have allergy?
10. Do you feel the following symptoms while you stay at home? (yes, often/yes, sometimes/no, never)
  (a) Fatigue
  (b) Heavy-headed
  (c) Headache
  (d) Dizziness
  (e) Difficulties in concentration
  (f) Itching, burning or irritation of eyes
  (g) Irritated, stuffy or runny nose
  (h) Dry or sore throat
  (i) Cough
  (j) Dry or flushed scalp or ears
  (k) Hand dry, itching, red skin
  (l) Memory impairment

## Appendix B

**Table A1:** Basic information about the apartments—Part 1.

No.	Year of Construction	Thermal Insulation	Floor	Heating Type	Total Floor Area (m^2^)	Floor area in the Living Room (m^2^)	Flooring	Furniture	Surface Finishing of Walls/Ceiling	Renovation in Less Than 30 Weeks
B1	1984–1993	no	>3rd	CH	≥50 < 100	18	L	particleboard	dispersive paint	no
B2	1955–1970	yes	>3rd	CH	≥50 < 100	16	L	particleboard	dispersive paint	no
B3	1971–1983	no	>3rd	CH	≥50 < 100	20	L	particleboard	dispersive paint	no
B4	1971–1983	yes	>3rd	CH	<50	16	L	wood	dispersive paint	no
B5	after 1993	yes	1st–3rd	CH	≥50 < 100	30	L	wood	dispersive paint	no
B6	1955–1970	yes	ground floor	CH	≥50 < 100	18	C	particleboard	dispersive paint	no
B7	1955–1970	yes	ground floor	CH	≥50 < 100	18	C	particleboard	dispersive paint	no
B8	1955–1970	no	1st–3rd	CH	≥50 < 100	35	L	particleboard	dispersive paint	no
B9	1971–1983	no	>3rd	CH	≥50 < 100	25	L	particleboard	dispersive paint	yes
B10	1984–1993	yes	>3rd	CH	<50	15	L	particleboard	dispersive paint	no
B11	1955–1970	yes	ground floor	CH	≥50 < 100	29	C	particleboard	dispersive paint	no
B12	1971–1983	yes	1st–3rd	CH	<50	26	L	particleboard	dispersive paint	no
B13	before 1955	no	1st–3rd	O	≥100 < 150	42	O	wood	dispersive paint	yes
B14	before 1955	no	1st–3rd	O	≥100 < 150	35	O	wood	dispersive paint	no
B15	1955–1970	yes	ground floor	CH	<50	12	C	particleboard	dispersive paint	no
B16	1984–1993	no	>3rd	CH	≥50 < 100	18	L	particleboard	dispersive paint	no
B17	1955–1970	yes	>3rd	CH	≥50 < 100	23	L	particleboard	dispersive paint	yes
B18	1984–1993	no	>3rd	CH	≥50 < 100	18	L	particleboard	dispersive paint	no

**Table A2:** Basic information about the apartments—Part 2.

No.	Number of Users	Pet	Traffic Density	Mould	Smoking Indoor	Fragrances	Plants	Cleaning Agents	Polishes/Waxes	Frequency of Cleaning	Opening Windows during Cleaning
B1	3–4	no	medium	no	yes	yes	8	yes	no	once a week	no
B2	≤2	yes	low	no	no	yes	15	yes	no	every 2–3 days	no
B3	≤2	yes	low	no	no	yes	2	yes	no	every 2 weeks	yes
B4	≤2	no	medium	yes	yes	yes	0	no	no	every 2–3 days	yes
B5	≤2	yes	low	no	yes	yes	4	yes	yes	every 2–3 days	yes
B6	≤2	no	medium	no	no	no	2	yes	no	every 2–3 days	yes
B7	3–4	no	medium	yes	no	no	8	yes	no	every 2–3 days	yes
B8	≤2	no	low	no	no	yes	4	yes	no	once a week	yes
B9	3–4	no	medium	yes	no	no	1	yes	yes	every 2–3 days	yes
B10	≤2	no	low	no	no	no	0	yes	no	once a week	yes
B11	≤2	no	medium	no	no	no	0	yes	no	once a week	no
B12	≤2	no	low	no	no	yes	2	yes	no	every 2–3 days	yes
B13	≤2	yes	medium	yes	no	no	6	yes	no	every day	no
B14	≤2	no	medium	no	yes	no	5	yes	no	once a week	no
B15	≤2	yes	low	no	no	yes	5	yes	no	once a week	yes
B16	>4	yes	low	no	yes	yes	0	yes	no	every day	yes
B17	3–4	no	high	no	no	no	0	yes	no	once a week	yes
B18	3–4	no	medium	yes	no	no	0	yes	no	once a week	yes

**Table A3:** Basic information about the family houses—Part 1.

No.	Year of Construction	Main Building Material	Thermal Insulation	Heating Type	Total Floor Area (m^2^)	Floor Area in the Living Room (m^2^)	Flooring	Furniture	Surface Finishing of Walls/Ceiling	Renovation in Less Than 30 Weeks	Garage	Attached Garage
D19	1955–1970	CO	polystyrene	SF	≥100 < 150	80	L	wood	dispersive paint	no	no	no
D20	after 1993	PC	-	SF	≥150	20	L	particleboard	dispersive paint	no	yes	yes
D21	1955–1970	CO	-	G	<50	12	L	particleboard	dispersive paint	yes	no	no
D22	before 1955	CS	polystyrene	G	≥50 < 100	20	O	particleboard	dispersive paint	no	yes	no
D23	before 1955	CS	-	G	≥150	25	L	particleboard	dispersive paint	no	yes	no
D24	before 1955	CS	polystyrene	G	≥50 < 100	15	L	particleboard	dispersive paint	no	yes	no
D25	1971–1983	BB	-	G	≥100 < 150	20	L	particleboard	dispersive paint	no	yes	yes
D26	after 1993	BB	polystyrene	SF	≥150	42	L	wood	dispersive paint	no	no	no
D27	1955–1970	PC	polystyrene	SF	≥150	23	L	particleboard	dispersive paint	no	yes	no
D28	after1993	BB	mineral	O	≥150	17	L	particleboard	dispersive paint	no	no	no
D29	1955–1970	BB	wool	G	≥100 < 150	17	C	particleboard	aluminium paint	no	yes	no
D30	1955–1970	CS	polystyrene	G	≥150	22	L	particleboard	dispersive paint	no	no	no
D31	after1993	PC	wood fibre	O	≥150	30	O	wood	dispersive paint	no	no	no
D32	after1993	BB	polystyrene	G	≥150	40	L	particleboard	dispersive paint	no	yes	no
D33	before 1955	BB	-	SF	≥100 < 150	18	C	particleboard	dispersive paint	no	no	no
D34	after1993	BB	polystyrene	G	≥150	89	L	wood	dispersive paint	no	yes	yes
D35	1971–1983	PC	polystyrene	G	≥150	16	L	particleboard	dispersive paint	no	yes	yes

**Table A4:** Basic information about the family houses—Part 2.

No.	Direct Entry from Garage to the House	Storage of Paints, Solvents, or Gasoline in the Garage	Number of Users	Pet	Traffic Density	Mould	Smoking Indoor	Fragrance	Plant	Cleaning Agents	Polishes/Waxes	Frequency of Cleaning	Opening Windows during Cleaning
D19	no	no	≤2	yes	low	no	yes	no	10	yes	no	every 2–3 days	yes
D20	yes	yes	3–4	no	low	no	yes	no	15	yes	no	every day	no
D21	no	no	≤2	no	low	no	no	no	0	yes	no	every 2–3 days	yes
D22	no	yes	≤2	no	low	no	no	yes	0	yes	yes	once a week	yes
D23	no	yes	>4	no	low	no	no	no	0	yes	yes	once a week	yes
D24	no	yes	3–4	no	low	no	no	yes	2	yes	yes	once a week	yes
D25	yes	yes	>4	no	low	yes	no	no	2	yes	no	once a week	no
D26	no	no	>4	no	low	no	no	no	4	yes	no	every day	no
D27	no	yes	3–4	no	low	yes	yes	yes	2	yes	yes	every 2–3 days	yes
D28	no	no	>4	no	medium	no	no	no	0	no	no	once a week	yes
D29	no	yes	≤2	no	low	yes	no	no	0	yes	no	once a week	no
D30	no	no	3–4	yes	medium	no	no	no	5	yes	no	every day	yes
D31	no	no	>4	no	low	no	no	no	18	yes	no	once a week	yes
D32	no	yes	3–4	no	medium	no	no	yes	11	yes	no	every day	yes
D33	no	no	>4	no	medium	yes	no	yes	7	yes	yes	every day	yes
D34	yes	yes	3–4	no	high	no	yes	no	5	yes	yes	every 2–3 days	yes
D35	yes	yes	3–4	yes	low	no	no	yes	5	yes	no	once a week	yes
